# Infection with the entomopathogenic nematodes *Steinernema* alters the *Drosophila melanogaster* larval microbiome

**DOI:** 10.1371/journal.pone.0323657

**Published:** 2025-05-16

**Authors:** Raymond Yau, Christina Pavloudi, Yingying Zeng, Jimmy Saw, Ioannis Eleftherianos

**Affiliations:** 1 Department of Biological Sciences, The George Washington University, Washington, DC, United States of America; 2 European Marine Biological Resource Centre-European Research Infrastructure Consortium (EMBRC-ERIC), Paris, France; University of Limpopo, SOUTH AFRICA

## Abstract

The fruit fly *Drosophila melanogaster* is a vital model for studying the microbiome due to the availability of genetic resources and procedures. To understand better the importance of microbial composition in shaping immune modulation, we can investigate the role of the microbiota through parasitic infection. For this, we use entomopathogenic nematodes (EPN) of the genus *Steinernema* which exhibit remarkable ability to efficiently infect a diverse array of insect species, facilitated by the mutualistic bacteria *Xenorhabdus* found within their gut. To examine the microbiome changes in *D. melanogaster* larvae in response to *Steinernema* nematode infection, *D. melanogaster* late second to early third instar larvae were exposed separately to *S. carpocapsae* and *S. hermaphroditum* infective juveniles. We have found that *S. carpocapsae* infective juveniles are more pathogenic to *D. melanogaster* larvae compared to the closely related *S. hermaphroditum*. Our microbiome analysis also indicates substantial changes in the size and composition of the *D. melanogaster* larval microbiome during infection with either nematode species compared to the uninfected controls. Our results serve as a foundation for future studies to elucidate the entomopathogenic-specific effector molecules that alter the *D. melanogaster* microbiome and understand the role of the microbiome in regulating insect anti-nematode immune processes.

## Introduction

The human microbiota includes more than 7,000 strains of over 1,000 species of bacteria, yeast, archaea, and viruses [1,2]. Substantial inter-individual variations in both the taxonomic composition of microbial communities and even gene content of the same microbial species have been previously reported [3,4]. One primary result from the international effort to fully characterize the human microbiome is the lack of a core human microbiota [5], with diet identified as the largest source of variation in the gut community [6,7]. Another significant factor predicting microbial assemblage is host genotype, with significant host-gut microbiota associations identified in humans [8,9], mice [10–12], and flies [13–15]. However, very little is known about the interaction between microbiota and pathogenic infection. A better understanding of host microbiome-pathogen interactions would increase effectiveness of microbiota-based therapies, enabling more targeted approaches to individualized treatment [16].

The fruit fly *Drosophila melanogaster* presents several advantages over mammalian models that enable finer dissection of host-gut microbiota interactions and how these regulate host defense against pathogenic organisms. Like mammalian models (e.g., mice), flies can be reared in the lab under controlled conditions, inbred, and crossed for many generations, but rearing flies is cheaper and faster. At the same time, flies have small, well-annotated genomes and sophisticated methods for genetically manipulating genes in specific tissues at discrete timepoints, often mediated by temperature-sensitive transgenes. There is also less redundancy in the fly genome compared to the mouse, allowing more straightforward interpretation of gene function and genetic pathways [17].

Despite the simpler, more experimentally tractable biology of *D. melanogaster*, the gut microbiota of flies in lab populations was believed to harbor considerably fewer species than in mammals [18], but more precise estimates have revealed a much more diverse gut community [19–21]. Public release of the human and fly genomes revealed that 70% of human disease genes have a homolog in flies, suggesting an ancient evolutionary origin to these pathways [22]. Now, with recent increases in the estimate of gut microbial taxa in *D. melanogaster*, there may be more overlap than expected with the human gut microbiome as well. As in humans, the fly gastrointestinal tract is partitioned into functionally distinct components, including the foregut, midgut, and hindgut. The peritrophic matrix in the midgut serves as a functional analog to the mammalian mucosal membrane, an important component of innate immunity and barrier integrity [23]. Also, like humans, the fly immune system maintains homeostasis of the gut microbiota, recognizing microbes using pattern recognition receptors such as Toll-like receptors that trigger cytokine responses [24,25]. Finally, the fly gut microbiota is known to influence several critical biological processes, including aging [26], locomotion [27], and metabolism [28], and behavior [28].

*Steinernema carpocapsae* is an entomopathogenic nematode (EPN) which rapidly infects and subdues a wide range of insects through immunosuppression during the initial stages of infection [29]. The infective juvenile (IJ) stage of *S. carpocapsae* nematodes forms an obligate mutualistic relationship with the bacterium *Xenorhabdus nematophila*. The IJs release the bacteria into the insect hemocoel after the parasites invade insects through natural openings. The *S. carpocapsae-X. nematophila* nemato-bacterial complex together with *D. melanogaster* serves as an excellent experimental model for understanding the interaction between EPN infection and host innate immune activity. *Steinernema hermaphroditum* is another EPN and the recently described Indian strain CS34 has been shown to be hermaphroditic when grown in vitro with healthy autonomously reproducing hermaphrodites and spontaneous functional males [30]. Due to their distinct life cycles and pathogenic effects on *D. melanogaster* larvae, *S. carpocapsae* and *S. hermaphroditum* are ideal for exploring their interaction with the insect microbiome during infection [31].

Understanding microbiome-pathogen interactions is key to revealing the role that certain microbes play in the host environment. Insights into how nematode infection influences the size and structure of the microbiome can lead to the identification of novel approaches to control parasitic diseases. Despite the importance of microbiome composition shaping the host immune system, there is currently limited information on the functional involvement of the microbiome in the innate immune response against parasitic nematode infection. Here, we challenge *D. melanogaster* wild type larvae with *S. carpocapsae* and *S. hermaphroditum* nematodes to determine changes in microbiome composition during different stages of infection. Findings from this work may be used to enhance EPN pathogenicity in the field by interfering with the microbiome of noxious insect pests.

## Materials and methods

### Fly stocks

Fly stocks were maintained and amplified on Drosophila diet (Fly Food B, LabExpress, Ann Arbor, MI) supplemented with yeast (Carolina Biological Supply, Burlington, NC). Drosophila melanogaster late second to early third instar larvae from parent strain Oregon-R (Bloomington Drosophila Stock Center; 33055) were kept in an incubator at 25 °C in a 12:12 hour light:dark photoperiodic cycle.

### Nematode stocks

*Steinernema hermaphroditum* strain CS34 nematodes were gifted from the lab of Paul Sternberg (California Institute of Technology, CA). The *Steinernema carpocapsae* (All) stock exists in our lab stock. All nematodes were cultured by infecting *Galleria mellonella* greater wax moth last instar caterpillars with nematode IJs in 6 cm Petri dishes (VWR, Bridgeport, NJ) and incubating the infected insects at 25 °C in a 12:12 hour light:dark photoperiodic cycle for 7 days before transferring them on a filtered paper plate trap filled with sterile pure water [32]. IJs selected for infection were within two weeks of collection date.

### Larval infection with nematodes

*Drosophila melanogaster* Oregon-R second to early third instar larvae were placed individually into the wells of a 96-well plate (ThermoFisher Scientific), which were previously filled with 100 μL of 1.25% agarose gel (Fisher Scientific). For larval infections with *S. hermaphroditum* or *S. carpocapsae*, each *D. melanogaster* larva was infected with 100 IJs of the respective entomopathogenic nematode suspended in 10 μL of sterile water. After, the 96-well plates were sealed with Masterclear real-time PCR film (Eppendorf, Enfield, CT), and holes were pierced for ventilation. Survival of infected larvae was monitored at 24-hour intervals and up to 144 hours. Survival rate was determined by dividing the number of living larvae at each time point by the total number of larvae tested. Larvae that escaped the plate were not considered in the survival rate analysis. For all survival experiments, control larvae were treated with 10 μL of pure water only. The survival experiments were repeated three times and each experiment involved at least thirty larvae [33].

### DNA extraction

DNA was extracted from live *D. melanogaster* larvae using the protocol in the Qiagen DNA extraction kit (Qiagen, Germantown, MD), according to the manufacturer’s instructions. For both nematode-infected and control experimental treatments, there were 60 larvae in each 96-well plate. DNA concentration was measured through Qubit 4 Fluorometer (Thermo Fisher Scientific, Waltham, MA). Each sample contained a minimum of 10 ng/μL DNA concentration in a total volume of 100 μL. The DNA concentration ranged from 30 ng/μL to 100 ng/μL with a purity range of 1.8 to 1.93.

### Library preparation and high-throughput sequencing

Amplicon libraries were prepared using Zymo Research’s Quick-16S kit with phased primers (341F – 806R) targeting the V3-V4 regions of the 16S rRNA gene. Following clean up and normalization, samples were sequenced on a P1 600cyc NextSeq2000 Flowcell to generate 2x301bp paired end (PE) reads. Quality control and adapter trimming was performed with bcl-convert1 (v4.2.4).

All the raw sequence files of this study were submitted to the European Nucleotide Archive (ENA) [34] with the study accession number PRJEB76541 (available at http://www.ebi.ac.uk/ena/data/view/PRJEB76541).

### Amplicon sequence analysis

DADA2 version 1.16 was used to filter and trim sequences, infer amplicon sequence variants (ASVs) and remove sequencing errors and chimeric sequences [35]. Taxonomy assignment was performed using SILVA release 138.1 [36]. For better comparison, subsequent analysis was conducted at the Species level and not at the ASV level. In addition, ASVs belonging to mitochondria and chloroplasts were removed before proceeding with the analyses.

Phyloseq package (version 1.42.0) was used to calculate alpha and beta diversity [37]. Alpha diversity was estimated using the Observed number of Species, Chao1, and abundance-based coverage estimator (ACE) indices [38,39]. Beta diversity was analyzed using Bray–Curtis distances [40] and visualized with nonmetric multidimensional scaling (nMDS); significance was assessed by permutational multivariate analysis of variance (PERMANOVA). Taxonomic groups that had significant differences in abundance among different groups (control *vs* infected) were identified by Linear Discriminant Analysis Effect Size analysis (LEfSe) [41] using the microbiomeMarker package (version 1.3.2) [42]. The Upset plot showing how many ASVs at the Species level were unique and how many were shared between the control and the infected groups was generated using UpSetR (version 1.4.0) [43] and ComplexUpset (version 1.3.3) [44,45]. Rarefaction curves were generated using the MicrobiotaProcess package (version 1.6.6) [46]. The aforementioned analyses were performed using R version 4.1.1 [47]. The R scripts used for the analyses of the sequences are provided in the GitHub repository (https://github.com/cpavloud/drosophila_microbiome).

### Statistical analysis

The *D. melanogaster* survival data were analyzed with a two tailed t test using the GraphPad Prism 9 software to estimate statistically significant differences between nematode infected larvae and uninfected controls. In addition, a Kaplan Meier curve was generated from the survival data using GraphPad Prism 9 software.

## Results

### Survival trend of *Drosophila melanogaster* second-third stage larvae following infection with entomopathogenic nematodes

**Fig 1:**
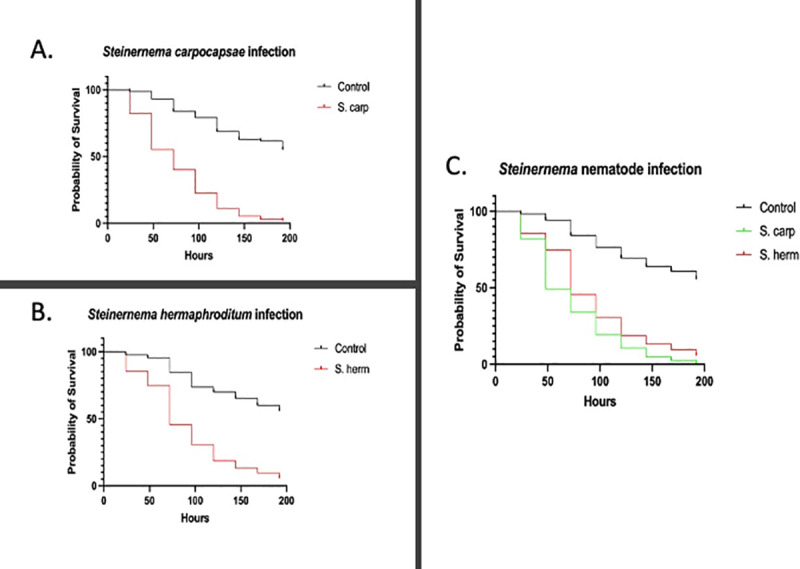
The effect of entomopathogenic nematode infection on *Drosophila melanogaster* larval survival. (A) Survival percentage of wild type *D. melanogaster* Oregon-R larvae over a course of 196 hours following parasitic nematode infection with *Steinernema carpocapsae.* The control larvae were treated with 10 μL of sterile pure water. (B) Survival percentage of wild type *D. melanogaster* Oregon-R larvae over a course of 196 hours following parasitic nematode infection with *Steinernema hermaphroditum.* The control larvae were treated with 10 μL of sterile pure water. A Log-rank test was used to calculate the statistically significant differences between the survival curves (*** P = 0.0001). (C) Survival percentage of wild type *D. melanogaster* Oregon-R larvae over a course of 196 hours following *Steinernema carpocapsae* or *Steinernema hermaphroditum* infection. A Log-rank test and Gehan-Breslow Wilcoxon test was used to calculate the statistically significant differences between the survival curves (* P = 0.0001).

For the *D. melanogaster* larval survival experiments, three timepoints were selected. The first timepoint selection was based on the initial decrease of survival, the second timepoint corresponds to approximately 50% mortality, and the final time point was selected when the survival was approximately 90%. Survival results show that at 24 hours, which serves as the first critical timepoint, there was 75% survival for *Drosophila melanogaster* larvae after infection with *S. carpocapsae* nematodes*,* a drastic decrease in survival at 48 hours (second critical timepoint) to over 50%, and 30% survival at the third critical timepoint of 72 hours (Fig 1A). However, after infection with *S. hermaphroditum* nematodes, there was 80% larval survival at 24 hours, 70% survival at 48 hours, and 45% survival at 72 hours (Fig 1B). Statistical analysis of the survival curves for *D. melanogaster* showed significant differences between the survival of nematode-infected larvae compared to uninfected control individuals (Fig 1A, P = 0.0001; Fig 1B, P = 0.0001; Fig 1C, P = 0.0001).

### Composition analysis of *Steinernema* nematode infection on the *Drosophila melanogaster* larval microbiome

**Fig 2:**
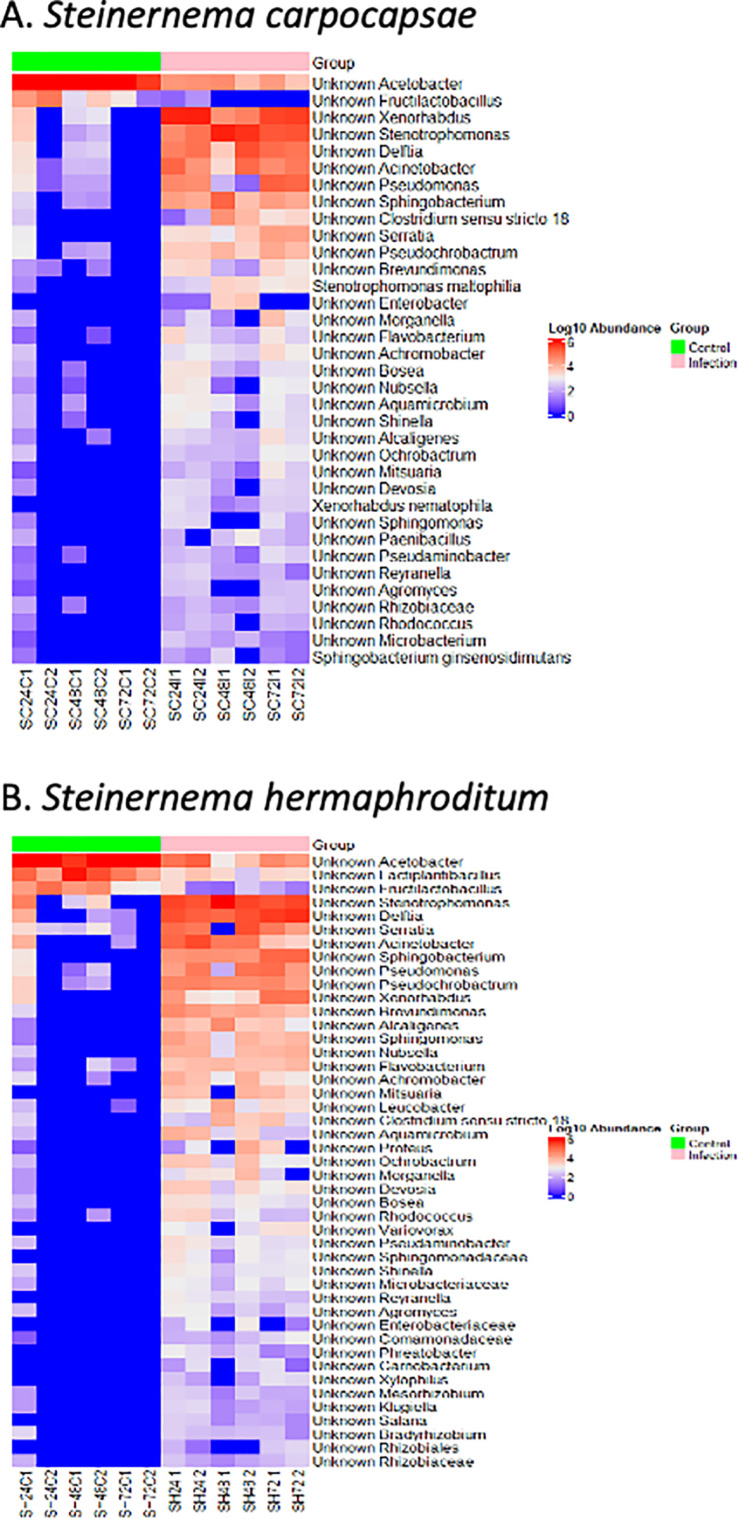
The effect of *Steinernema* entomopathogenic nematode infection on *Drosophila melanogaster* larval microbiome composition. Heatmaps comparing the differentially abundant bacterial species identified in the *D. melanogaster* larval microbiome between control and (A) *Steinernema carpocapsae* and (B) *Steinernema hermaphroditum* infected samples. The color key on the right indicates Log10 Abundance with the color red highlighting higher abundance, whereas the color blue defines low abundance.

*Steinernema carpocapsae* displayed higher pathogenicity than *S. hermaphroditum* (Fig 1, P = 0.0001), which led to the hypothesis that the *S. carpocapsae* infected *D. melanogaster* larvae would potentially display more pronounced compositional changes in the microbiome than *S. hermaphroditum* infected individuals. To explore the compositional microbiome alterations in *D. melanogaster* larvae following entomopathogenic nematode infection, heatmaps were generated from the LefSe analysis to visualize the significant changes in bacterial species based on the treatment (control *vs* infected), regardless of the time point (Fig 2).

*Acetobacter* stably colonizes the gut of wild type *D. melanogaster* and interacts with the *Lactobacillus* species to influence fly fat content and genes involved in lactate and acetoin metabolism [48]. In the *S. hermaphroditum* infections, the *Acetobacter, Fructilactobaullius,* and *Lactiplantibacillus* species were highly abundant in the control group but sharply decreased in the infected groups (Fig 2A). The same pattern follows in the *S. carpocapsae* infections, where *Acetobacter* species were highly abundant in the control group but undergone a substantial decrease in the nematode infected group. The *Fructilactobacillus* species was also nearly abolished in the *D. melanogaster* larval microbiota when comparing the control group to the infected group (Fig 2B).

*Xenorhabdus* spp. is the symbiotic bacterium of the entomopathogenic nematodes *Steinernema* spp. These insect pathogenic Gram-negative bacteria quickly multiply within the insect host and are strongly present in the infected group for both *S. carpocapsae* and *S. hermaphroditum* infections when comparing infected and uninfected control groups. Infection with either *Steinernema* parasitic nematode species leads to an increase in the *Stenotrophomonas*, *Delftia*, *Acinetobacter*, *Pseudochrobactrum*, *Sphingobacterium*, and *Clostridium sensu stricto* species when compared to the control group (Fig 2A and 2B).

Unique to the *S. carpocapsae* infection, the *Serratia* and *Stenotrophomonas maltophilia* were markedly enhanced in abundance in comparison to the control group. Moderate increases to the microbiota composition of the infected group included *Morganella, Achromobacter, Flavobacterium, Bosea, Aquamicrobium, Shinella, Alcaligenes, Paenibacillus, Xenorhabdus nematophila, Reyranella,* and *Microbacterium* (Fig 2A). *Steinernema hermaphroditum* infection resulted in a higher bacterial diversity including: *Brevundimonas, Alcaligenes, Nubsella, Achromobacter, Flavobacterium, Mitsuaria, Aquamicrobium, Leucobacter, Ochrobactrum, Devosia, Morganella, Rhodococcus, Bosea, Pesudaminobacter, Sphingomonadacease, Variovorx, Shinella, Microbacteriaceae, Reyranella, Agromyces, Phreatobacter, Enterobacteriaceae, Comamondadaceae, Carnobacterium, Xylophilus, Salana, Klugiella, Mesohizobium, Rhizobiaceae, Rhizobiales, Camelimonas,* and *Kaistia* species (Figs 2B and 5).

**Fig 3:**
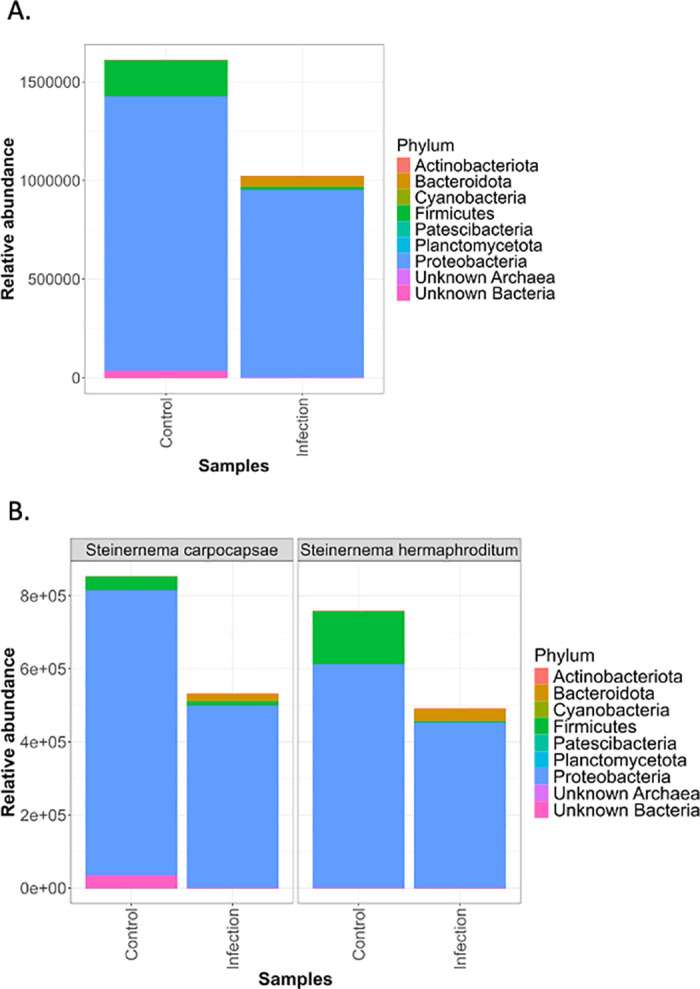
The relative size of the *Drosophila melanogaster* larval microbiome after *Steinernema* infection. (A) All phyla combined from both *Steinernema carpocapsae* and *Steinernema hermaphroditum* infection treatments. The illustrated bar graphs comprise the control group (larvae treated with pure water) and infection group (larvae treated with entomopathogenic nematodes). (B) The relative abundance determined by *Steinernema carpocapsae* or *Steinernema hermaphroditum* infection treatments.

**Fig 4:**
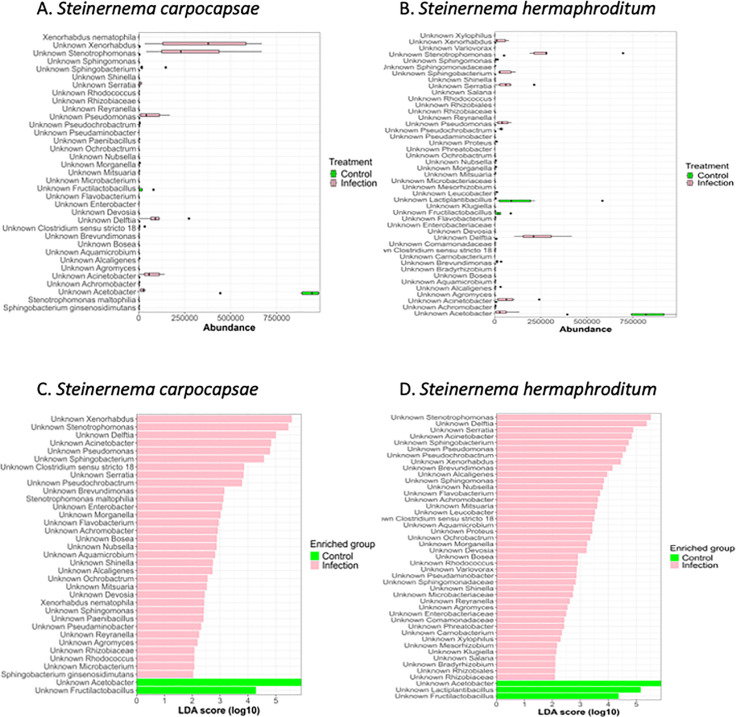
Differentially enriched bacterial species by nematode infection group in the *Drosophila melanogaster* larval microbiome. (A) The abundance of bacterial species during *Steinernema carpocapsae* infection in comparison to uninfected control. Through linear discriminant analysis (LDA) effect size (LEfSe), the *S. carpocapsae* infection group was categorized based on the treatment in order to identify the differentially enriched taxa in the control and in the infected larva. (B) The abundance of bacterial species during *Steinernema hermaphroditum* infection in comparison to uninfected control. The *S. hermaphroditum* infection group was categorized based on the treatment in order to identify the differentially enriched taxa in the control and in the infected samples. (C) Histogram of the LDA scores computed for the differentially abundant taxa discovered in the *D. melanogaster* larval microbiome during *S. carpocapsae* infection. The linear discriminant analysis demonstrates the differentially abundant species among the treatment group. The control treatment indicates LDA score of 4 ≤ for *Acetobacter, Burkholderia-Caballeronia,-Parabukholderiam* and *Fructilactobacillus*. (D) Histogram of the LDA scores computed for the differentially abundant taxa discovered in the *D. melanogaster* larvae microbiome during *S. hermaphroditum* infection. The linear discriminant analysis demonstrates the differentially abundant species among the treatment group. The control treatment indicates LDA score of 4.5 ≤ for *Acetobacter, Lactiplantibacillus* and *Fructilactobacillus*.

### Changes in the size of the *Drosophila melanogaster* larval microbiome during parasitic nematode infection

After determining the effects of each *Steinernema* nematode species on *D. melanogaster* larval microbiome composition, the relative size of the microbiome was analyzed. The bacterial community associated with both the infected and the control samples consisted mainly of Proteobacteria; however, Alphaproteobacteria were highly abundant in control samples while Gammaproteobacteria were highly abundant in the infected samples. The overall abundance for bacterial phyla was reduced in infected groups by nearly 50% compared to the control (Fig 3A). Furthermore, extrapolating specific phyla from the relative abundance analysis, control groups had consistently higher abundance of Firmicutes (Fig 3A). This overall trend was prevalent in both *S. carpocapsae* and *S. hermaphroditum* infection treatment groups, with the infected groups having a stark decrease in the overall abundance and the control group having nearly twice the relative abundance (Fig 3B). Infected groups demonstrated an increase in *Bacteroidota* and striking reduction in *Firmicutes* (Fig 3B). The reduction in the microbiome size could be indicative of the entomopathogenic nematode strategy to target commensal microbes in order to defeat the insect host.

### Enriched bacterial species by treatment groups in the *Drosophila melanogaster* larval microbiome

Based on the *D. melanogaster* larval survival results, it was speculated that *S. carpocapsae* infection may have a stronger effect on the microbiome than *S. hermaphroditum* infection (Fig 1C). The LefSe analysis on the abundance of bacterial species in nematode infection groups also revealed that *S. carpocapsae* infection primarily induced specific changes in prevalence of few bacterial taxa and *S. hermaphroditum* infection caused a general alteration to a multitude of bacterial taxa (Fig 2).

Examining the LefSe analysis for bacterial species, *S. carpocapsae* infection groups were primarily rich in abundance for *Xenorhabdus* and *Stenotrophomonas* species, while the uninfected controls were rich in *Acetobacter* species (Fig 2A). In the *S. carpocapsae* infected group, 35 biomarkers were identified out of which two were enriched in the control group and 33 in the infection group (S1 and S2 Tables). When the timepoint was included as a subgroup, 25 biomarkers were identified, with two from the control group and 23 from the infection group (S1 and S2 Tables). The LefSe abundance analysis for *S. carpocapsae* infection highlighted the enrichment of *Xenorhabdus, Stenotrophomonas, Pseudomonas, Delftia, Acinetobacter,* and *Acetobacter*, while *Fructilactobacillus* and *Acetobacter* were enriched in the uninfected control group (Fig 4A). The LefSe abundance analysis for *S. hermaphroditum* infection indicated the enrichment of *Xenorhabdus, Stenotrophomonas, Serratia, Pesudomonas, Delftia, Sphingobacterium, Acinetobacter,* and *Acetobacter*, while *Fructilactobacillus* and *Acetobacter* were enriched in the control group (Fig 4B). Furthermore, the linear discriminant analysis for *S. carpocapsae* infection demonstrated that both *Xenorhabdus* and *Stenotrophomonas* had a LDA score of ≤ 5, indicating an exponential increase of their presence in the microbiota (Fig 4C). Observations of other bacterial species present in the infection treatment also had a LDA score of ≤ 2 (Fig 4C); however, the abundance was lower compared to *Xenorhabdus* and *Stenotrophomonas*. Through the linear discriminant analysis for *S. hermaphroditum* infection, *Stenotrophomonas, Sphingobacterium, Acinetobacter, Delftia, Xenhorbadus,* and *Pseudomonas* had a LDA score of ≤ 4.5 (Fig 4D). Noticeably, *Pseudochrobactrum* and *Brevundimonas* also had a LDA score of ≤ 4.5 but held relatively lower abundances (Fig 4B). The control group in *S. hermaphroditum* had a similar trend to the *S. carpocapsae* control treatment, where the *Acetobacter* and *Lactiplantibacillus* species were abundant and had a LDA score of ≤ 4.5 (Fig 4D). The *S. hermaphroditum* infection group presented a different infection pattern, where 42 out of the total 45 biomarkers were enriched in the infection group and when the timepoint was included as a subgroup, 38 out of the total 40 biomarkers were enriched in the infection group (S1 and S2 Tables).

### Nonmetric multidimensional scaling (nMDS) plot depicting the similarities between the samples

When all the samples were considered, grouping based on treatment, i.e., control and infection, was statistically significant (PERMANOVA: R^2^ = 0.60, p = 0.001) while grouping based on the nematode species was not (PERMANOVA: R^2^ = 0.04, p = 0.44). When samples of the *S. carpocapsae* infection group only were selected, grouping based on treatment was statistically significant (PERMANOVA: R^2^ = 0.60, p = 0.004) while grouping based on the sampling time point, i.e., 24, 48 and 72 hours, was not (PERMANOVA: R^2^ = 0.15, p = 0.541). When samples of the *S. hermaphroditum* infection group only were selected, grouping based on treatment was more pronounced (PERMANOVA: R^2^ = 0.72, p = 0.002), but again grouping based on the sampling point was not significant (PERMANOVA: R^2^ = 0.08, p = 0.771) (S1 Fig).

### Comparing bacterial species by entomopathogenic nematode treatment

**Fig 5:**
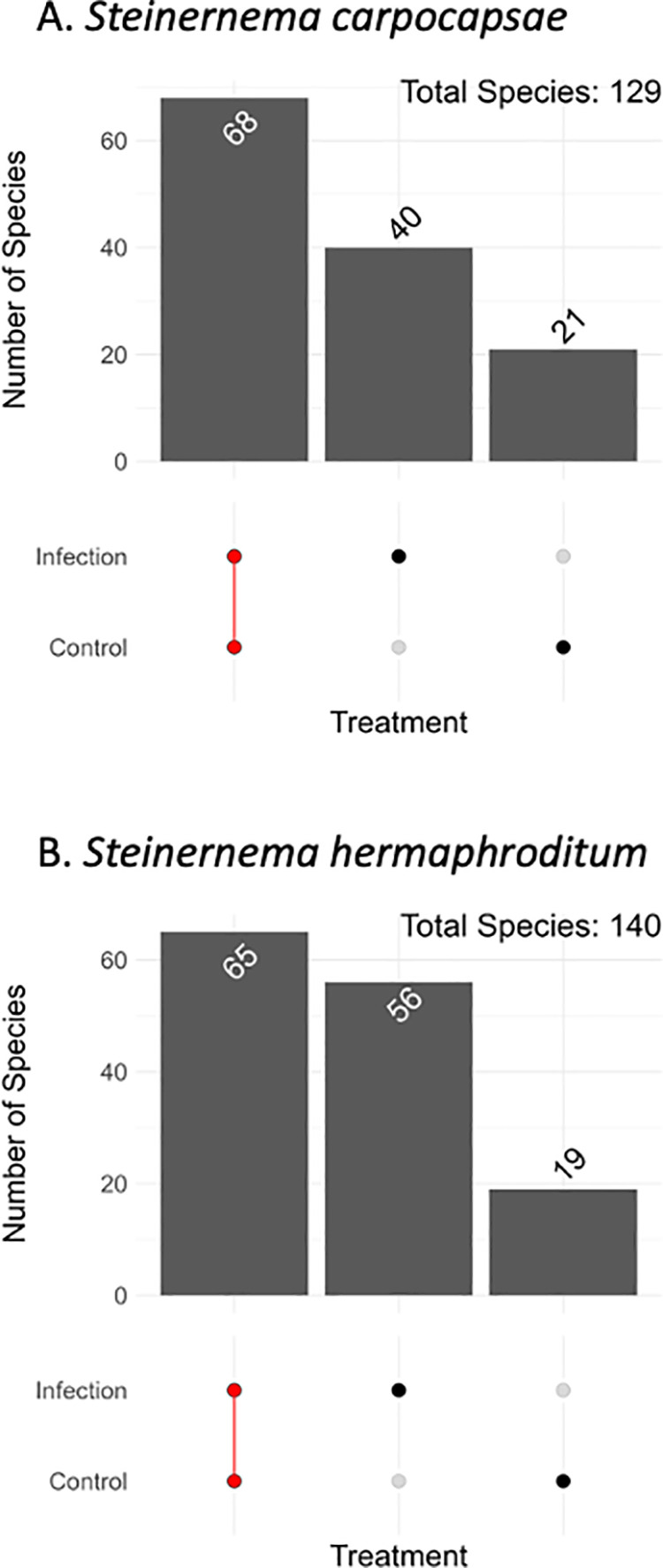
Comparison of the amplicon sequence variants (ASVs) between *Steinernema* infection treatment groups in the *Drosophila melanogaster* larvae. (A) The number of amplicon sequence variants (ASVs) for *D. melanogaster* larvae treated with *Steinernema carpocapsae*. (B) Number of amplicon sequence variants (ASVs) for *Drosophila melanogaster* larvae treated with *Steinernema hermaphroditum.*

According to the upset plots, in the *S. carpocapsae* samples, the control and the infection group shared 68 ASVs identified at the species level in common, while 40 were found only in the infected samples and 21 were found only in the control samples (Fig 5A). In the *S. hermaphroditum* samples, 65 ASVs identified at the species were shared between the control and the infected samples, 56 ASVs were found only in the infected samples and 19 ASVs were unique in the control samples, suggesting that larvae infected with *S. hermaphroditum* contain higher number of identified species (Fig 5B).

### Alteration in the bacterial community of the *Drosophila melanogaster* larval microbiome following nematode infection

**Fig 6:**
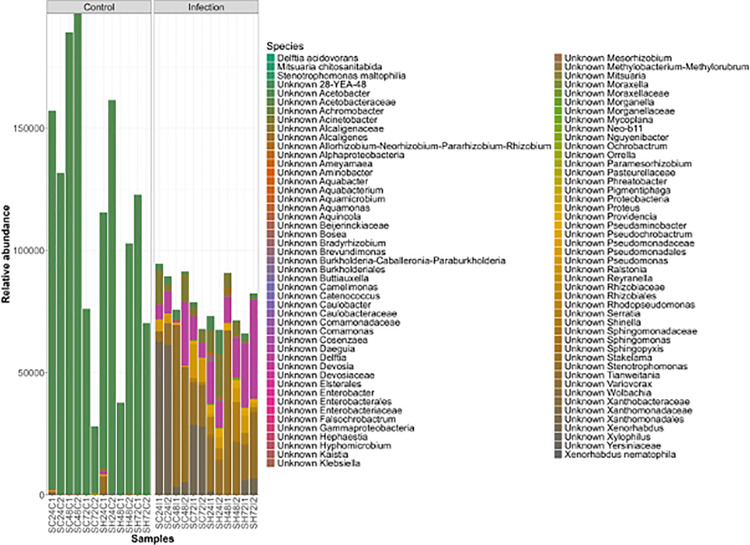
Bar chart identifying species belonging to the Proteobacteria phylum. Relative abundances of species belonging to the phylum Proteobacteria in the uninfected control and nematode infected samples.

Comparison of the bacterial communities associated with the treatment group shows that the nematode infected samples included the class Bacteroidia (phylum Bacteroidota), while it was nearly absent in the control samples (S2A Fig). Bacteroidia are significant clinical pathogens and typically present in anaerobic infections [49], and generally aid in maintaining a complex and beneficial relationship with the host when retained in the gut. However, if these bacteria escape this environment, they can cause detrimental effects such as bacteremia and abscess formation in multiple body sites [49]. Further studies are needed to elucidate the role of each Bacteroidia species to better understand which species are beneficial or detrimental (S2A Fig). When examining the Firmicutes communities in both infected and uninfected control groups, they were dominantly present in the control samples (S2B Fig). These results suggest that *Steinernema* nematodes induce significant reduction in Firmicutes in infected larvae, leading to the host’s demise (S2 Fig). Gammaproteobacteria is dominant in the bacterial community associated with nematode infected samples, while Alphaproteobacteria is dominant in uninfected controls (S2C Fig). Proteobacteria species significantly differed between the uninfected control and infected samples, with the control samples having a restricted diversity (Fig 6). The control samples primarily consisted of *Acetobacter* species from the Alphaproteobacteria class, while the infected samples consisted of a range of species such as *Delftia acidovorans*, *Stenotrophomonas maltophila*, and others from the Gammaproteobacteria class (Fig 6).

### Estimating microbial species richness in the *Drosophila melanogaster* larval microbiome

**Fig 7:**
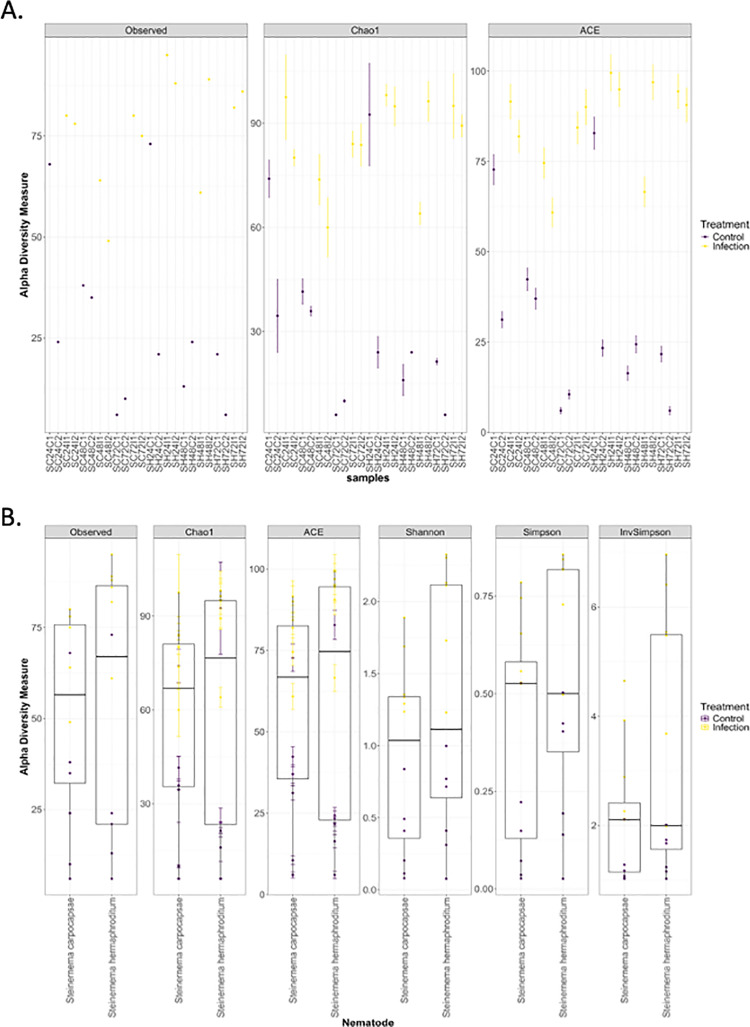
Alpha diversity metrics for bacterial communities between treatment groups. (A) Using observed number of ASVs, Chao1, and ACE indices to estimate microbial diversity. (B) Boxplots showing the distribution of alpha diversity metrics values for *Drosophila melanogaster* larvae infected by either *Steinernema carpocapsae* or *S. hermaphroditum* nematodes.

**Fig 8:**
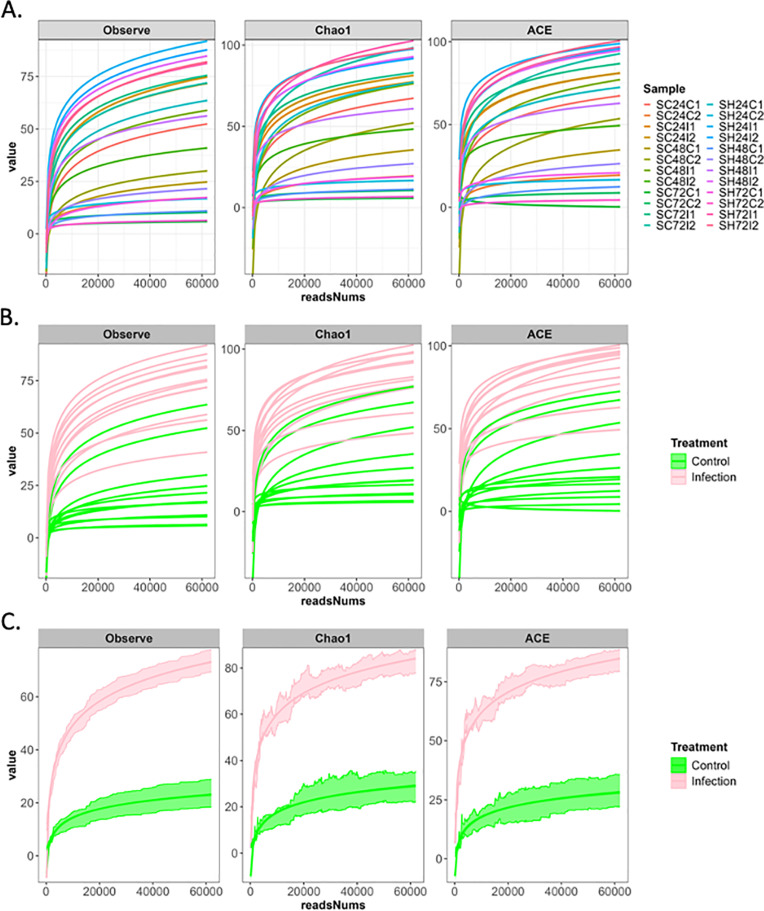
Rarefaction curve for both control and infected groups. The horizontal axis represents the sequencing depth of each sample, and the vertical axis shows the alpha diversity index (Observed ASVs, Chao1 index, and ACE index). (A) The rarefaction curve for each sample. (B) The rarefaction curve for each sample colored by the treatment group. (C) The rarefaction curve for each treatment group with standard error of the mean.

Using observed number of Species, Chao1, and ACE indices, we find that in most cases, the values of the indices were higher in the nematode infected samples as compared to the uninfected control samples (Figs 7A, 8). The median values of alpha diversity indices were similar between the two nematode infected samples in almost all cases, except in the case of the Observed number of species, Chao1 and ACE indices where *S. hermaphroditum* values were higher (Fig 7B). The rarefaction curves for the control samples reached a plateau sooner than the infected samples, indicating that, although enough sequences have been acquired for each sample to be compared fairly in subsequent analyses, the infected samples are more diverse (Fig 8).

## Discussion

The microbiome plays an important role in the physiological functions and health in a variety of organisms. Beneficial symbiotic microbes which comprise the microbiota contribute to improving the quality of life of the host [50]. *Drosophila melanogaster* serves as the optimal model for investigating host-microbe and microbe-microbe interactions in invertebrates due to the convenient manipulation of microbial composition [50].

The amenable system of *D. melanogaster* offers insight to the relationship between microbiota composition and microbes, with a low-diversity microbiota primarily dominated by five species in the genera *Acetobacter* and *Lactobacillus* [51]. Axenic *D. melanogaster* without *Acetobacter* and/or *Lactobacillus* displayed delay larval development and hyperglycemia, but the reintroduction of these species allows for partial or complete restoration of conventional traits in *D. melanogaster* [51]. Entomopathogenic nematodes are lethal to insects and are used in biocontrol strategies against agricultural pests and disease vectors. The entomopathogenic nematode *Steinernema carpocapsae* holds a mutualistic relationship with *Xenorhabdus nematophila* bacteria and demonstrates higher pathogenicity than *S. hermaphroditum*. The latter holds a mutualistic relationship with the *X. griffiniae* bacteria and goes through four larval stages (J1-J4) before they mature into adults. During infection, the entomopathogenic nematode releases the symbiotic bacterial partner into the host once the worm enters the hemocoel.

The release of bacteria in the insect host alters the survival following the secretion of toxins and virulence factors which target insect tissues. In our experiments, *D. melanogaster* larvae in the second-third instar stage were infected with either *S. carpocapsae* or *S. hermaphroditum* infective juveniles. The survival of the *D. melanogaster* larvae for the two different *Steinernema* nematodes demonstrates distinct trends. Both survival trends had an altered rate upon entomopathogenic nematode infection when compared to control uninfected larvae treated with pure water only, however *S. carpocapsae* demonstrated higher pathogenicity than *S. hermaphroditum*. Previous studies indicated that *S. hermaphroditum*/*X. griffiniae* and *S. carpocapsae*/*X. nematophila* may utilize distinct infection strategies during *D. melanogaster* larval infection [31]. Also, the *D. melanogaster* immune response is likely to be regulated differently by *S. hermaphroditum* compared to *S. carpocapsae.* Infection of *D*. *melanogaster* larvae with *S. hermaphroditum* fails to induce the expression of readout genes in the two NF-κB pathways, Imd and Toll, as well as the JNK pathway [31]. However, previous work has demonstrated that injection of *S. carpocapsae* excreted-secreted products into *D. melanogaster* adult flies upregulates the expression of antimicrobial peptide-encoding genes and reduces the activity of the phenoloxidase enzyme in their hemolymph [52]. The suppression of immune signaling activation in larvae infected with *S. hermaphroditum* is potentially promoted by *X. griffiniae*. It was previously demonstrated that *X. nematophila* infection induces antibacterial peptides in the insect host but inhibits phagocytosis and the melanization response [53].

A previous work examined the bacterial communities in *Tenebrio molitor* larvae following a fatal *Steinernema* nematode infection [54]. To induce infection, three species of *Steinernema* nematodes were used, namely *S. carpocapsae, S. feltiae*, and *S. weiseri*. The authors found that *Xenhorhabdus* does not dominate the bacterial community in the insect host, and nematode infections alter the composition of the bacterial community, although substantial variability was noted. When exploring the compositional microbiome alterations in *D. melanogaster* larvae after entomopathogenic nematode infection, *S. hermaphroditum* produced a more profound composition alteration than *S. carpocapsae*. *Lactobacillus* serves as a probiotic and *Acetobacter* is critical in nutritional regulation and primarily dominates the microbiota of fruit flies, which were both demonstrated to be consistently reduced in both infections. The two genera of bacteria are important for normal larval development and nutritional regulation [51]. As both *Steinernema* entomopathogenic nematodes hold a mutualistic relationship with a distinct *Xenorhabdus* bacterial species, there are overlapping introductions of detrimental bacteria. For example, *Acinetobacter*, a Gram-negative bacterium causes infections in the blood and urinary tract and certain strains are antibiotic resistant [55,56], surged in abundance for both *Steinernema* infections. One of the less known drug-resistant Gram-negative bacteria *Stenotrophomonas maltophilia* leads to challenging infections through its powerful virulence factors which form a biofilm; *S. maltophilia* has been characterized as a global opportunistic pathogen infecting a range of organs and tissues and associated with respiratory infections in humans [57]. This bacterial pathogen became prominent in our microbiome analysis. *Xenorhabdus* was present in all nematode infected samples and acted as positive control confirming that entomopathogenic nematode infection occurred successfully. Other species predominantly appeared in both types of nematode infections included: *Delftia*, a Gram-negative bacillus which is typically nonpathogenic but in severe situations can cause pneumonia and bacteremia [58], and *Pseudochrobactrum*, a Gram-negative bacterial pathogen [59], which has been found to be innocuous in *Galleria mellonella* larvae [60]. *Delftia* has also been proposed as a probiotic species against entomopathogenic fungal infection in the fall armyworm (*Spodoptera frugiperda*) [61] and mosquito (*Aedes aegypti*) [62], Beneficial bacterial species were also introduced to potentially fend off the nematode infection, such as *Clostridium sensu stricto*, an anaerobe which metabolizes various compounds including carbohydrates, amino acids, alcohols, and purines [63]. Gut microbiota dysbiosis can induce further pathogenesis of parasitic infection, however studies in post-stroke depression events revealed the intake of insulin increases the abundance of *C. sensu stricto* in the intestines to promote nutrition uptake [64]. Other potential beneficial bacteria candidates include *Sphingobacterium* and *Pseudomonas* [65], however further studies are needed to elucidate if they are beneficial to the host. The composition alteration revealed these genera were simply introduced and were not present in the natural microbiota of *D. melanogaster*, indicating these bacteria could be harmful. There are cases where these bacterial genera were demonstrated to be beneficial in plants, however in humans can be a different situation. *Pseudomonas* is an opportunistic Gram-negative bacterium which contains lipopolysaccharides in its outer membranes, which serves as a virulence factor that influences development of psychopathology, resulting in anxiety and depression [66]. *Sphingobacterium* is a Gram-negative bacterium which is ubiquitous and rarely causes infection in humans, however it has the potential to cause fatal infections and bacteremia [67]. *Sphingobacterium* has also been found to suppress fungal growth in infected *Delia antiqua* larvae [68]. The appearance of many bacterial species during *S. hermaphroditum* infection reveals several culprits known for promoting detrimental immune effects, more so than *S. carpocapsae*. However, many of the bacterial species found during *S. hermaphroditum* infection remain unknown and could not be further classified.

The *D. melanogaster* larval microbiome composition was significantly altered, but another notable observation was the reduction in the overall microbiome size and abundance of bacteria genera. A common trend was a significant abundance of *Firmicutes* in the microbiome of uninfected control *D. melanogaster* larvae suggesting the potential involvement of these bacteria in systemic immunity to protect the host against infection [69]. Mammals and other organisms have evolved in environments occupied by microbes, especially the intestinal microbiota which supports development, metabolism, behavior, and defense against infection. Although it is still poorly understood how members of the microbiota control immunity systemically, *Firmicutes* is a great candidate for elucidating how this symbiotic relationship can regulate the immune system without conferring detrimental effects, such as inflammation and organ damage [69]. Recent work in wild-type mice with well-defined communities of gut microbes revealed that lysozyme undergoes mucosal processing to create *Firmicutes* which then releases peptidoglycan to disseminate and prime systemic innate antibacterial defenses [70]. These peptidoglycans can persist systematically for a long period of time and allow for greater systemic immunity control. There is a fine balance between mechanisms of tolerance and resistance for *Firmicutes* role in systemic immunity, however if host-microbiota mutualism breaks down, then the systemic influence becomes pathogenic and leads to devastating tissue inflammation, organ damage, and cachexia [69]. This is a possible explanation for the reduction in *Firmicutes* in the microbiome when the *D. melanogaster* larvae are infected with entomopathogenic nematodes, a friend turned foe. As previously classified, *Bacteriodota* is a phylum consisting of three large classes of Gram-negative, anaerobic or aerobic, and rod-shaped bacteria. Members of the genus *Bacteriodota* are potential colonizers of the gut microbiome and major players for sustaining the microbial food web of the gut [71]. When examining the *D. melanogaster* larval microbiome composition following infection with either *S. carpocapsae* or *S. hermaphroditum*, the introduction of many Gram-negative bacteria was detrimental to the insect host. However, the microbiome is still poorly understood as the genus *Bacteriodota* also contains gut commensals which serve many roles including protection from pathogens and allocation of nutrients to other microbial residents [71]. Using 16S rRNA sequencing, we were only able to classify known species and even then, it was harder to identify between closely related bacterial species. Although a parasitic infection was facilitated, there is a large spike in the abundance of *Bacteriodota* which makes it difficult to discount if there were commensal bacteria species being introduced.

Closer examination of the enriched bacterial species after entomopathogenic nematode infection reveals that the two *Steinernema* nematode species rely on separate specific bacterial species. In *S. carpocapsae* infections, there were two primary enriched bacterial species: *Xenorhabdus,* the symbiotic bacteria to *S. carpocapsae* and *Stenotrophomonas*, an unfamiliar drug-resistant Gram-negative bacterium. In *S. hermaphroditum* infections, the enriched species were relatively even in abundance however it was a multitude of bacterial species including *Xenorhabdus, Stenotrophomonas, Sphingobacterium, Pseudomonas, Delftia,* and *Acinetobacter*. Although samples grouped based on nematode species were not significant, the enriched bacterial species and their abundance should be further investigated to depict the variation in composition. However, examination of the enriched taxa in the infection groups suggests that *S. hermaphroditum* contains 41 out of 47 species whereas *S. carpocapsae* contains 21 out of 47 species. *Steinernema hermaphroditum* is a new entomopathogenic nematode that was identified from soil samples in Indonesia’s Moluccan islands [72]. Comparing it to *S. carpocapsae*, a well-annotated nematode that originates from mole crickets in Uruguay and Argentina, creates an excellent case study. There has been relatively little research on the microbiome of either nematode species, with only rudimentary studies addressing the possible effects of *Steinernema* nematodes on the composition of the microbiota [73]. Future research, however, will examine the possibility that some microbes could be obtained via the intermediary host—in this case, *Galleria mellonella*—that is employed to culture the nematode species. A typical approach to investigating the microbiome is to use amplicon sequencing, a type of High-Throughput Sequencing (HTS) of 16S rRNA gene [74]. Amplicon sequence variants (ASVs) are an alternative to operational taxonomic units (OTUs) for analyzing microbial communities [75]. ASVs are commonly used because they reflect a more refined level of taxonomy and do not cluster sequences according to a distance-based threshold. The *S. carpocapsae* and the *S. hermaphroditum* infected groups include more ASVs compared to the uninfected control group. The identification of genetic differences between the infected and control groups suggests changes in gene expression possibly leading to changes in the makeup of the microbiota. The entomopathogenic nematodes significantly altered the abundance of the microbiome, and the ASVs demonstrate there is potentially different gene expression impacting gene regulations. Previous studies elucidated how environmental pollutants can not only shift the gut microbial structure but also alter triglyceride- and cholesterol-regulating genes [76]. Drugs such as esketamine also impacted intestinal flora and through differential expression analysis, 301 genes were significantly upregulated and 106 genes significantly downregulated [76]. But there is currently unknown how entomopathogenic nematodes can similarly affect the alteration of gene expression.

## Concluding remarks and future directions

Here we find that the *D. melanogaster* larval microbiome composition is significantly modified in response to infection with the EPN species *S. carpocapsae* and *S. hermaphroditum*. Also, our results reveal an overall reduction in size and abundance of bacteria genera of the *D. melanogaster* microbiome. Future efforts could expand to *D. melanogaster* larvae following infection with other EPN species including the well-studied *Heterorhabditis bacteriophora* or *H. indica*. The production of germ-free *D. melanogaster* larvae without *Acetobacter* and/or *Lactiballius* would also contribute to elucidating how the remaining flora interacts with the *Photorhabdus* spp. symbiotic bacteria that are vectored by *Heterorhabditis* spp. nematodes. Another interesting route would be to generate axenic nematodes to separate the effect of the nematode vector alone on the *D. melanogaster* microbiome. EPN are viable in the absence of their symbiotic while retaining their pathogenic properties towards insects [32]. The generation of *D. melanogaster* larvae containing a single or multiple gut bacterial species would further determine the inter- and intra-species interactions in response to the nematode-bacteria complex and each pathogen (nematode or bacteria) independently. Finally, the results from similar experiments performed on *D. melanogaster* adult flies would help us understand how changes in microbiome composition upon nematode infection or challenge with specific EPN effector molecules interferes with innate immune system regulation at different developmental stages, and whether the microbiome composition is retained from the larval to the adult stage.

## Supporting information

S1 Fig NMDS plot depicting the similarities between all samples. Samples were statistically compared through different groupings: treatment, nematode species, and together.
(TIFF)

S2 Fig Bar plots comparing dominant bacterial phyla after parasitic nematode infection in the *Drosophila melanogaster* larval microbiome. (A) Bar chart comparing the relative abundance of Bacteroidota phylum in all samples. (B) Bar chart comparing the relative abundance of Firmicutes in all samples. (C) Bar chart comparing the relative abundance of Proteobacteria in all samples.
(TIFF)

S1 Table Enriched taxa in the nematode infection groups, as derived from LefSe. (DOCX)

S2 Table Enriched taxa in the control groups, as derived from LefSe. (DOCX)
