# Supplementary figures and images for: Infection with the entomopathogenic nematodes Steinernema alters the Drosophila melanogaster larval microbiome
Source: PLoS One. 2025 May 16;20(5):e0323657. doi: 10.1371/journal.pone.0323657 (PMC12084044; doi:10.1371/journal.pone.0323657)

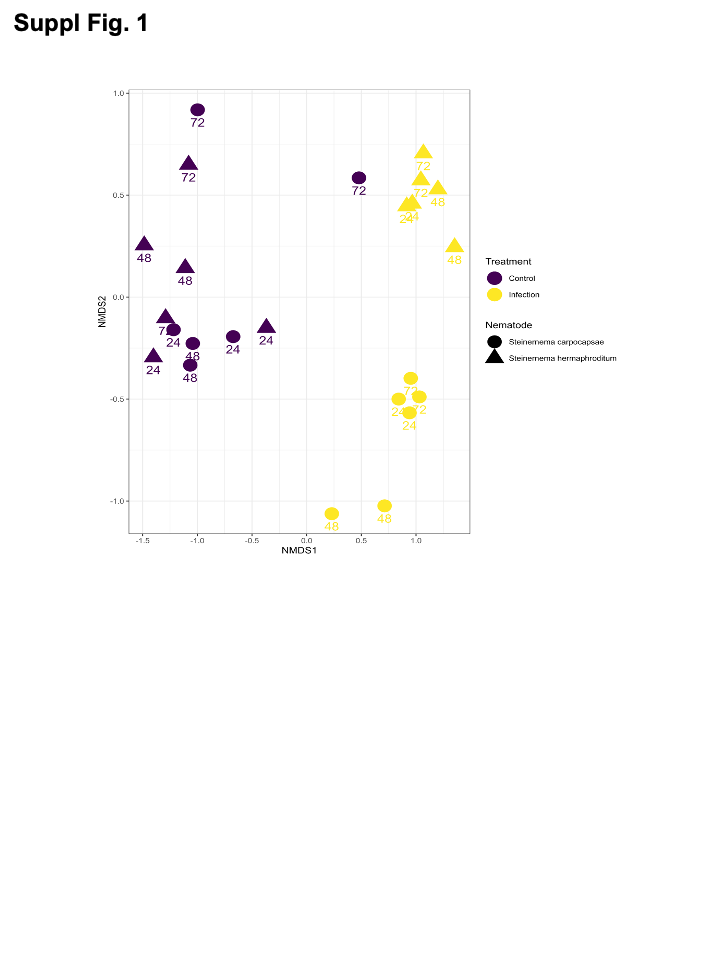

Supplement: S1 Fig — Samples were statistically compared through different groupings: treatment, nematode species, and together. (TIFF) [file pone.0323657.s001.tiff]

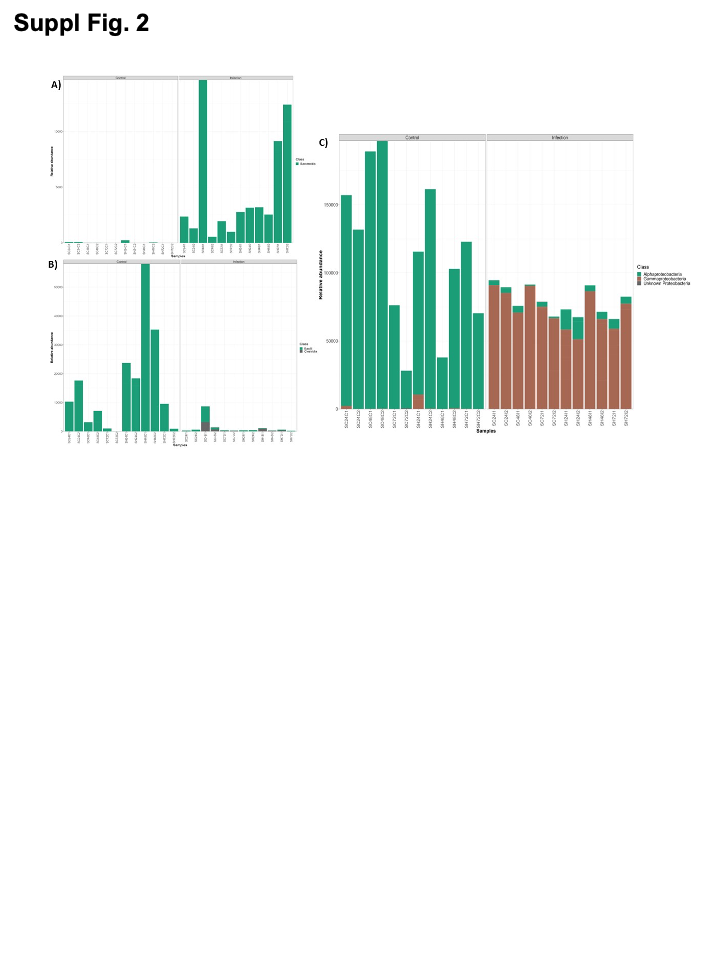

Supplement: S2 Fig — (A) Bar chart comparing the relative abundance of Bacteroidota phylum in all samples. (B) Bar chart comparing the relative abundance of Firmicutes in all samples. (C) Bar chart comparing the relative abundance of Proteobacteria in all samples. (TIFF) [file pone.0323657.s002.tiff]
