# Supplementary material for: Infection with the entomopathogenic nematodes Steinernema alters the Drosophila melanogaster larval microbiome
Source: PLoS One. 2025 May 16;20(5):e0323657. doi: 10.1371/journal.pone.0323657 (PMC12084044; doi:10.1371/journal.pone.0323657)
Supplement: S1 Table — (DOCX) [file pone.0323657.s003.docx]

**Suppl. Table 1: Enriched taxa in the nematode infection groups, as derived from LefSe.**

|  | ***Steinernema carpocapsae*** | | ***Steinernema hermaphroditum*** | | |
| --- | --- | --- | --- | --- | --- |
| **Taxa** | **Treatment** | **Treatment (Timepoint)** | **Treatment** | | **Treatment (Timepoint)** |
| *Achromobacter* | * | * | * | * | |
| *Acinetobacter* | * | * | * |  | |
| *Agromyces* | * |  | * | * | |
| *Alcaligenes* | * | * | * | * | |
| *Aquamicrobium* | * | * | * | * | |
| *Bosea* | * | * | * | * | |
| *Bradyrhizobium* |  |  | * |  | |
| *Brevundimonas* | * | * | * | * | |
| *Carnobacterium* |  |  | * | * | |
| *Clostridium sensu stricto 18* | * |  | * | * | |
| *Comamonadaceae* |  |  | * | * | |
| *Delftia* | * | * | * | * | |
| *Devosia* | * | * | * | * | |
| *Enterobacter* | * |  |  |  | |
| *Enterobacteriaceae* |  |  | * | * | |
| *Flavobacterium* | * | * | * | * | |
| *Klugiella* |  |  | * | * | |
| *Leucobacter* |  |  | * | * | |
| *Mesorhizobium* |  |  | * | * | |
| *Microbacterium* | * | * |  |  | |
| *Microbacteriaceae* |  |  | * | * | |
| *Mitsuaria* | * | * | * | * | |
| *Morganella* | * |  | * | * | |
| *Nubsella* | * |  | * | * | |
| *Ochrobactrum* | * | * | * | * | |
| *Paenibacillus* | * | * |  |  | |
| *Phreatobacter* |  |  | * | * | |
| *Proteus* |  |  | * | * | |
| *Pseudaminobacter* | * | * | * | * | |
| *Pseudochrobactrum* | * | * | * | * | |
| *Pseudomonas* | * |  | * | * | |
| *Reyranella* | * | * | * | * | |
| *Rhizobiaceae* | * |  | * |  | |
| *Rhizobiales* |  |  | * |  | |
| *Rhodococcus* | * | * | * | * | |
| *Salana* |  |  | * | * | |
| *Serratia* | * | * | * | * | |
| *Shinella* | * |  | * | * | |
| *Sphingobacterium* | * | * | * | * | |
| *Sphingobacterium ginsenosidimutans* | * |  |  |  | |
| *Sphingomonadaceae* |  |  | * | * | |
| *Sphingomonas* | * |  | * | * | |
| *Stenotrophomonas* | * | * | * | * | |
| *Stenotrophomonas maltophilia* | * | * |  |  | |
| *Variovorax* |  |  | * | * | |
| *Xenorhabdus* | * | * | * | * | |
| *Xenorhabdus nematophila* | * | * |  |  | |
| *Xylophilus* |  |  | * | * | |
