# Supplementary material for: Infection with the entomopathogenic nematodes Steinernema alters the Drosophila melanogaster larval microbiome
Source: PLoS One. 2025 May 16;20(5):e0323657. doi: 10.1371/journal.pone.0323657 (PMC12084044; doi:10.1371/journal.pone.0323657)
Supplement: S2 Table — (DOCX) [file pone.0323657.s004.docx]

**Suppl. Table 2: Enriched taxa in the control groups, as derived from LefSe.**

|  | ***Steinernema carpocapsae*** | | ***Steinernema hermaphroditum*** | |
| --- | --- | --- | --- | --- |
| **Taxa** | **Treatment** | **Treatment (Timepoint)** | **Treatment** | **Treatment (Timepoint)** |
| *Acetobacter* | * | * | * | * |
| *Fructilactobacillus* | * | * | * |  |
| *Lactiplantibacillus* |  |  | * | * |
